# Personal views of aging in midlife and older age: the role of personality

**DOI:** 10.3389/fpsyg.2024.1437232

**Published:** 2024-10-09

**Authors:** Elena Carbone, Enrico Sella, Diletta Signori, Erika Borella

**Affiliations:** Department of General Psychology, University of Padova, Padova, Italy

**Keywords:** views of aging, subjective age, awareness of age-related change, personality traits, midlife and old age

## Abstract

**Introduction:**

Personal views of aging (VoA) reflect individuals’ perceptions, attitudes, and expectations regarding their aging selves. The present cross-sectional study was aimed at examining whether personality traits, as defined by the Big Five model, are associated with different VoA concepts related to both subjective age and awareness of age-related gains and losses in midlife and older age.

**Materials and methods:**

A sample of 224 participants aged 46–85 years reported their felt age and completed the Awareness of Age-Related Change (AARC) questionnaire, assessing perceptions of age-related gains (AARC-Gains) and losses (AARC-Losses) in various functioning domains, as well as the short version of the Big Five Inventory.

**Results:**

Linear regression models showed that Openness contributed to explain youthful subjective age. Extraversion explained higher AARC-Gains scores, whereas Emotional Stability, along with younger chronological age and perceiving better self-rated health, contributed to explaining lower AARC-Losses scores.

**Discussion:**

These findings confirm the relationship between personality traits and personal VoA. They further suggest that such an association varies depending on the VoA measure considered. They underscore the importance of considering personality among those individual characteristics capable of shaping personal VoA, with implications for the development of tailored interventions and the understanding of the underlying mechanisms linking personal VoA to health and longevity outcomes in midlife and older age.

## 1 Introduction

Personal views of aging (VoA) refer to individuals’ perceptions, attitudes, and expectations related to one’s aging process (Shrira et al., 2022) and are an integral part of adults’ experience of growing older. The well-established link between personal VoA and health, well-being, and longevity outcomes (Sabatini et al., 2020b, 2023; see Sabatini et al., 2020a; Westerhof et al., 2023 for meta-analyses) unfolds a renewed and increasing interest in this construct. A growing body of research has shown that holding positive personal VoA relates to better mental and physical outcomes in adulthood and older age (Sabatini et al., 2020a; Pinquart and Wahl, 2021) through three potential pathways, namely psychological (e.g., adaptive coping strategies and self-regulation, positive self-concept), behavioral (e.g., engagement in health enhancing, preventive behaviors), and physiological (e.g., adaptive physiological responses to stressors) (Sabatini et al., 2024; Westerhof et al., 2023).

Personal VoA is an umbrella term encompassing a variety of related yet also sufficiently distinct concepts (Brothers et al., 2017, 2019; Kornadt et al., 2020; Shrira et al., 2022), such as subjective or felt age, attitudes toward one’s aging, and the more recent conceptualization of awareness of age-related change (AARC). Felt age is usually operationalized by asking individuals how old they feel, and the discrepancy between an individual’s felt age and actual chronological age is used as the expression of self-perception of one’s aging (e.g., Montepare, 2009; Diehl et al., 2014). Felt age is therefore seen as psychologically distancing oneself from one’s “true” age and age peers and captures personal VoA in a rather general, unidimensional way, without explicit reference to individuals’ specific personal aging experiences (Brothers et al., 2017; Diehl et al., 2014; Sabatini et al., 2024). Attitudes toward one’s aging represent individuals’ general cognitive and affective evaluations of their aging process (Diehl et al., 2014) as well as their expectations about their experience of being older adults. AARC, on the other hand, refers to an individual’s awareness that their behavior, level of performance, or ways of experiencing life have changed as a consequence of growing older (e.g., Diehl and Wahl, 2010). The AARC captures positive (AARC-Gains) and negative (AARC-Losses) subjective evaluations of one’s aging across various behavioral and life domains of functioning (e.g., physical, cognitive, socio-emotional; Diehl and Wahl, 2010). AARC-Gains and AARC-Losses have been theoretically conceptualized as two distinct subcomponents of the same AARC construct, which could operate in parallel but not totally independently (Diehl and Wahl, 2010; Sabatini et al., 2020a). Such a gain-loss factor structure of AARC, which has been also empirically supported (Brothers et al., 2019), aligns with the essential lifespan developmental proposition that aging is characterized by positive and negative development (Baltes, 1987) and therefore that gains and losses represent separate aspects of the perceived aging experience (Brothers et al., 2019; Diehl and Wahl, 2010; Diehl et al., 2014; Sabatini et al., 2020a).

Given the multidimensionality of VoA and their implications in various life domains, efforts are increasing in terms of understanding whether individuals’ VoA may depend on individual characteristics. Recent, renewed, and well-established theoretical frameworks of VoA propose a whole host of sociodemographic, biological/health-related, and psychological factors as potential antecedents capable of influencing individuals’ perceptions and experiences of their aging, and among them is also personality (Diehl and Wahl, 2010; Diehl et al., 2014; Shrira et al., 2022; Sabatini et al., 2024).

According to the dominant Big Five model (Costa and McCrae, 1992), personality refers to an individual’s consistent pattern of thoughts, feelings, and actions, which can be comprehensively described along the five distinct, broad dimensions of Extraversion, Conscientiousness, Neuroticism, Agreeableness, and Openness. Personality traits represent individual characteristics that remain quite stable across the lifespan (Costa et al., 2000, 2019) and are well known to influence relevant life outcomes (e.g., quality of interpersonal relationships; Roberts et al., 2007), cognitive functioning (Carbone et al., 2019; Curtis et al., 2015), physical and psychological health, well-being, and longevity (Friedman and Kern, 2014; Ozer and Benet-Martinez, 2006; Terracciano et al., 2008). These basic attitudinal characteristics provide a coherent and consistent cognitive, emotional, and behavioral frame of reference through which individuals select or avoid certain situations and environments (Costa et al., 2000, 2019) and can shape how people think about aging and one’s aging process (Kornadt et al., 2019). In line with attitudinal approaches in social psychology (Kandler et al., 2014) and lifespan developmental psychology (Baltes, 1987; see also Costa et al., 2019), personality traits can in fact influence the way people dynamically experience, perceive, and adapt to age-related changes by predisposing individuals, from a behavioral viewpoint, to adopt—or not—health-enhancing behaviors and active lifestyles (e.g., engagement in leisure, physical and social activities; adherence to treatments), which are key factors for successful/healthy aging (Borella et al., 2023). Playing a role in one’s perception of self (e.g., Ozer and Benet-Martinez, 2006), they can also influence the way individuals interpret, evaluate, and appraise everyday situations and experiences arising throughout life and with aging (Hubley and Hultsch, 1994; Rupprecht et al., 2019).

Notwithstanding the flourishing literature on the interplay between individual characteristics, VoA, and health and longevity outcomes (see Westerhof et al., 2023), personality’s role seems to have received little attention. There is indeed only some few and initial evidence linking personality to personal VoA concepts in adulthood and older age. Some studies have found youthful subjective age linked to Extraversion, reflecting an inclination toward positive emotions, sociability, and an active, engaged lifestyle and to Openness, or a propensity for intellectual curiosity and a liberal attitude (e.g., Canada et al., 2013; Hubley and Hultsch, 1994, 1996; Stephan et al., 2012; Weiss et al., 2019). Though consistent, the contribution of Extraversion and Openness to explaining felt age is usually very modest [2% of explained variance for Openness in Weiss et al. (2019); 5.4% and of explained variance for Extraversion and 4% of explained variance for Openness in Hubley and Hultsch (1996)]. Neuroticism, a tendency to experience distress, anxiety, and negative emotions, has been consistently related to less positive attitudes toward aging whereas Extraversion, Agreeableness (reflecting cooperativeness and altruism), and Conscientiousness (a tendency to be responsible, organized, hard-working, and goal-directed) are linked to more positive attitudes toward one’s aging (e.g., Bryant et al., 2016; Miche et al., 2014; Dunsmore and Neupert, 2022; Kornadt et al., 2019). Only four studies to date have examined the associations between personality traits and AARC (Dunsmore and Neupert, 2022; Rupprecht et al., 2019; Wahl et al., 2013; Wettstein et al., 2022). Their results showed Neuroticism is consistently associated with high AARC-Losses (e.g., *r* = 0.46 in Wahl et al., 2013; *r* = 0.48 in Rupprecht et al., 2019; Dunsmore and Neupert, 2022) whereas high AARC-Gains were found linked to Extraversion (*r* = 0.14 in Wahl et al., 2013), Openness (e.g., *r* = 0.17 in Rupprecht et al., 2019; *r* = 0.44 in Dunsmore and Neupert, 2022), and Conscientiousness (e.g., *r* = 0.14 in Rupprecht et al., 2019).

Taken together, these findings suggest that certain personality traits are associated with personal VoA concepts in adulthood and older age, depending on the personality dispositions and VoA measures considered. However, previous studies neither always considered the contribution of all personality traits (e.g., Hubley and Hultsch, 1994, 1996; Miche et al., 2014; Wahl et al., 2013; Weiss et al., 2019) nor systematically jointly considered the complex and multidimensional nature of VoA or its facets. Further, the extent to which personality might help explain the recent concept of AARC with its gains and losses domains is less understood.

Therefore, the aim of the present study was to further examine and confirm the relationship between the Big Five personality traits and various personal VoA facets, namely felt age, AARC-Gains and AARC-Losses, in midlife and older age.

According to previous evidence (e.g., Hubley and Hultsch, 1994, 1996; Stephan et al., 2012), we expected Extraversion and Openness to be associated with feeling younger than one’s chronological age. We further explored the associations between personality traits and AARC-Gains and AARC-Losses. Because Emotional Stability (the opposite of Neuroticism), Extraversion, Openness, and Conscientiousness might not only predispose individuals to engage healthier aging lifestyles but also elicit heightened sensitivity and more positive evaluations and reactivity toward positive aging experiences (Friedman and Kern, 2014; Terracciano et al., 2008; Rupprecht et al., 2019), we could expect them, in line with previous limited evidence (Dunsmore and Neupert, 2022; Rupprecht et al., 2019; Wahl et al., 2013; Wettstein et al., 2022), to be associated with a high awareness of age-related gains and a low awareness of age-related losses.

## 2 Method

### 2.1 Participants

The study involved 224 community-dwelling adults and older adults aged 46–85 years (75% females). All participants were native Italian speakers and were recruited by word of mouth, volunteering for the study.

Inclusion criteria were as follows: (i) no history of major physical or mental health issues as assessed through a semi-structured interview (De Beni et al., 2008); (ii) a Montreal Cognitive Assessment-BLIND score ≥ 17 (MoCA-BLIND; Wittich et al., 2010; i.e., no signs of neurocognitive disorder); and (iv) a Geriatric Depression Scale score ≤ 5 (GDS; Yesavage et al., 1982; i.e., no sign of major depressive symptoms).

Table 1 shows the descriptive statistics of participants’ sociodemographic characteristics and screening measures.

**Table 1:** Descriptive statistics of participants’ sociodemographic characteristics, screening measures and measures of interest.

	Min-max	*M*	SD
Sociodemographic characteristics and screening measures			
Age (years)	46–85	61.54	9.87
Education (years)	4–26	11.87	3.86
Montreal cognitive assessment-BLIND	17–22	19.42	1.54
Geriatric depression scale	0–5	1.92	1.57
Self-rated health	2–5	3.73	0.64
Personal views of aging			
AARC-Gains	37–117	84.81	15.19
AARC-Losses	26–92	54.22	14.71
Felt age	−0.41 to 0.32	−0.13	0.13
Personality traits			
Agreeableness	3–10	6.67	1.45
Conscientiousness	4–10	8.24	1.28
Emotional Stability	2–10	6.28	1.75
Extraversion	2–10	6.65	1.52
Openness	2–10	6.88	1.67

AARC, awareness of age-related change.

### 2.2 Materials

#### 2.2.1 Personal views of aging

*Awareness of Age-Related Change* (AARC; adapted from Brothers et al., 2019). This scale comprises 50 items, 25 assessing AARC-Gains and 25 assessing AARC-Losses. Out of the 25 items on each scale, five items address each of the AARC life and behavioral domains (health/physical functioning, cognitive functioning, interpersonal relationships, socio-cognitive and socio-emotional functioning, lifestyle engagement). Participants rated the extent to which each item applied to them on a 5-point Likert scale (from 1 = *not at all* to 5 = *very much*). The dependent variables were the scores for AARC-Gains and AARC-Losses, calculated by summing the 25 items falling into the respective subscales (max = 125; Cronbach’s alpha for AARC-Gains = 0.89 and AARC-Losses = 0.90). Higher scores indicate higher AARC-Gains and AARC-Losses.

*Felt age*. Participants were asked to provide their subjective age with a single-item question: “*Please indicate the age that you feel from 0 to 120 years.*” Proportional discrepancy scores (dependent variable) were calculated for each participant as a measure of felt age to control for the various effects of chronological age (Debreczeni and Bailey, 2021) as follows: subjective age – chronological age/chronological age, with negative scores corresponding to feeling younger than one’s chronological age^1^.

#### 2.2.2 Personality traits

*The 10-item Big Five Inventory* (Guido et al., 2015). It consists of 10 items that assess the five major personality traits: Agreeableness (e.g., “I see myself as someone who is generally trusting”), Conscientiousness (e.g., “I see myself as someone who does a thorough job”), Emotional Stability (e.g., “I see myself as someone who is relaxed and handles stress well”), Extraversion (e.g., “I see myself as someone who is outgoing and sociable”), and Openness (e.g., “I see myself as someone who has an active imagination”), with acceptable reliability (Spearman-Brown coefficients >0.50). Participants were asked to indicate their agreement with each statement on a 5-point Likert scale (1 = *strongly disagree*; 5 = *strongly agree*). The dependent variables were obtained by summing the scores on the two items expressing each of the five major personality traits.

#### 2.2.3 Control variables

Chronological age, years of education, gender (0 = female, 1 = male) and self-rated health were controlled due to their associations with both personal VoA and personality (e.g., Costa et al., 2019; Löckenhoff et al., 2008; Roberts et al., 2007; Sabatini et al., 2023). As for self-rated health, participants were asked to rate their physical and psychological health on a 5-point Likert scale (1 = *very poor*; 5 = *very good*) with two *ad-hoc* questions (“*How do you rate your overall physical health?*” and “*How do you rate your overall psychological health?*”). A composite score expressing overall self-rated health was calculated and considered, with higher scores corresponding to better perceived health.

### 2.3 Procedure

After giving their written informed consent, all participants attended an individual session lasting about 90 min, conducted remotely (via Zoom or Skype platforms) by a trained experimenter, to complete a series of tasks and questionnaires in the following order: a semi-structured interview that included questions on sociodemographic information, felt age and physical and psychological health, the MoCA-BLIND, the 10-item Big Five Inventory, the AARC, and the GDS.

### 2.4 Statistical analyses

To investigate the relationships between personality traits and personal VoA, linear regression model (LM) analyses were conducted separately for felt age, AARC-Gains, and AARC-Losses scores. A model comparison approach was used, starting from a null model (intercept only) to a full model (i.e., including all predictors).

Given that sociodemographic characteristics (age, gender, education) and self-rated health have been related to both personal VoA and personality (e.g., Costa et al., 2019; Löckenhoff et al., 2008; Roberts et al., 2007; Sabatini et al., 2023), these variables were controlled. An age^2^ term was included to test for non-linear associations between chronological age and personal VoA (e.g., Wettstein et al., 2022). Therefore, we ran a null model (m0: ′y ~ intercept), followed by a model including sociodemographic characteristics and self-rated health (m1: ′y ~ intercept + chronological age + chronological age^2^ + education + self-rated health). Subsequently, the full model was computed, including sociodemographic characteristics, self-rated health, and personality traits (m2: ′y ~ intercept + chronological age + chronological age^2^ + education + self-rated health + Agreeableness + Conscientiousness + Emotional Stability + Extraversion + Openness)^2^.

All the LMs were run using the lm() function in R software (R Core Team, 2019). Each model was compared to the previous one using the Akaike Information Criterion (AIC; Akaike, 1973). The most plausible model for each considered variable was the one with the lowest AIC value (Burnham et al., 2011). To account for issues regarding multiple testing, the alpha level was set to *p* ≤ 0.016.

## 3 Results

**Figure 1:**
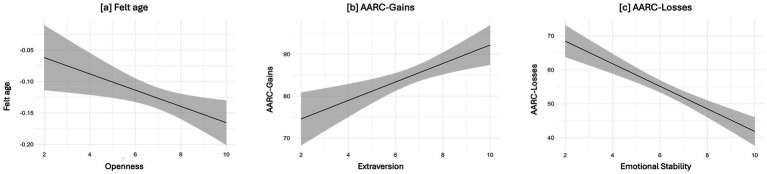
Plots of the associations between felt age and Openness (a), AARC-Gains and Extraversion (b), and AARC-Losses and Emotional Stability (c).

Table 1 shows the descriptive statistics of the outcomes of interest. Information on the model comparison approach and the best model selection for each VoA measure are shown in Table 2. Results from the best model for each VoA measure of interest are reported in Table 3. Figure 1 shows the plots of significant associations between personal VoA facets and personality traits that emerged. Additional information on the matrix of correlations among the measures of interest are available in Supplementary Table S1.

**Table 2:** Model comparison results for each measure of interest.

Measure of interest	Model	Predictors	AIC	*R* ^2^	Δ*R*^2^	adj *R*^2^
Felt age	m0	Intercept	−280.8185			
	m1	Intercept+age + age^2^ + gender+education+ self-rated health	−284.4734	0.059		
	**m2**	**Intercept + age + age**^**2**^ **+ gender + education + self-rated health + Agreeableness + Conscientiousness + Emotional Stability + Extraversion + Openness**	**−286.5958**	**0.108**	**0.049**	**0.066**
AARC-Gains	m0	Intercept	1857.484			
	m1	Intercept+age + age^2^ + gender+education+ self-rated health	1858.635	0.038		
	**m2**	**Intercept + age + age**^**2**^ **+ gender + education + self-rated health + Agreeableness + Conscientiousness + Emotional Stability + Extraversion + Openness**	**1851.165**	**0.111**	**0.073**	**0.069**
AARC-Losses	m0	Intercept	1843.025			
	m1	Intercept+age + age^2^ + gender+education+ self-rated health	1779.515	0.279		
	**m2**	**Intercept + age + age**^**2**^ **+ gender + education + self-rated health + Agreeableness + Conscientiousness + Emotional Stability + Extraversion + Openness**	**1762.208**	**0.362**	**0.083**	**0.332**

The most parsimonious, best-fit model, i.e., with the lowest AIC, selected for each measure of interest (felt age, AARC-Gains and AARC-Losses) appears in bold. AARC, awareness of age-related change; age, chronological age.

**Table 3:** Results (estimates and standardized solutions) of the best models for felt age and AARC-Gains and Losses scores.

	Felt age					AARC-Gains					AARC-Losses				
	Est	CI		*B*	*p*	Est	CI		*B*	*p*	Est	CI		*B*	*p*
Intercept	−0.191	−0.144	−0.093		<0.001	86.818	83.800	89.836		<0.001	55.366	52.891	57.841		<0.001
Age	**−0.002**	**−0.004**	**0.001**	**−0.198**	**0.016**	0.140	−0.107	0.388	0.091	0.264	**0.345**	**0.142**	**0.549**	**0.232**	**0.001**
Age^2^	0.000	−0.0002	0.0001	−0.025	0.749	−0.011	−0.032	0.009	−0.088	0.268	−0.001	−0.019	0.015	−0.013	0.838
Gender	0.013	−0.053	0.026	−0.044	0.505	−3.481	−8.161	1.199	−0.098	0.144	−3.966	−7.804	−0.129	−0.116	0.042
Education	0.002	−0.006	0.002	−0.062	0.398	−0.533	−1.101	0.034	−0.135	0.065	−0.355	−0.821	0.109	−0.093	0.133
Self-rated health	0.015	−0.043	0.013	−0.075	0.305	2.152	−1.261	5.566	0.090	0.215	**−5.824**	**−8.623**	**−3.024**	**−0.254**	**<0.001**
Agreeableness	0.005	−0.016	0.005	−0.062	0.340	0.170	−1.176	1.517	0.016	0.803	−0.562	−1.667	0.541	−0.055	0.316
Conscientiousness	0.002	−0.015	0.010	−0.026	0.684	0.577	−0.950	2.106	0.048	0.457	1.212	−2.466	0.040	−0.105	0.057
Emotional Stability	0.009	−0.019	0.0008	−0.125	0.073	1.281	0.082	2.480	0.147	0.036	**−2.054**	**−3.037**	**−1.071**	**−0.244**	**<0.001**
Extraversion	0.006	−0.004	0.017	0.073	0.266	**2.201**	**0.897**	**3.506**	**0.220**	**0.001**	−0.649	−1.719	0.419	−0.067	0.232
Openness	**0.013**	**−0.023**	**0.003**	**−0.172**	**0.012**	−0.289	−1.506	0.928	−0.031	0.639	−0.706	−1.704	0.291	0.080	0.164

AARC, awareness of age-related change. Significant predictors appear in bold.

### 3.1 Felt age

The full model (m2) emerged as the most plausible (*R*^2^ = 0.11; see Table 2). Compared with the model including only sociodemographics and perceived health, personality traits additionally explained 4.9% of the variance for felt age (see Table 2). Chronological age and Openness emerged as the significant predictors for felt age, indicating that older individuals with higher Openness were the ones who feel younger than their chronological age (see Table 3 and Figure 1a).

### 3.2 AARC-Gains

The full model (m2) emerged as the most plausible (*R*^2^ = 0.11; see Table 2). Compared with the model including only sociodemographics and perceived health, personality traits additionally explained 7% of the variance for AARC-Gains scores (see Table 2). Extraversion emerged as a significant predictor of AARC-Gains, indicating that more extraverted individuals reported higher awareness of age-related gains (see Table 3 and Figure 1b).

### 3.3 AARC-Losses

The full model (m2) emerged as the most plausible (*R*^2^ = 0.36; see Table 2). Compared with the model including only sociodemographics and perceived health, personality traits additionally explained an 8% of the variance for AARC-Losses scores (see Table 2). Higher Emotional Stability, along with younger chronological age and better self-rated health, predicted lower AARC-Losses scores (see Table 3 and Figure 1c).

## 4 Discussion

The present study further explored the associations between the Big Five personality traits and personal VoA in midlife and older age with a cross-sectional design. Alongside the prominent concept of subjective age, awareness of age-related gains and losses was also considered to account for a more comprehensive, multidimensional evaluation of individuals’ self-perception of their aging.

Our results, in line with previous evidence (Hubley and Hultsch, 1994, 1996; Weiss et al., 2019), showed that personality contributed to explain, albeit modestly, subjective age: particularly, individuals with high Openness felt younger than their chronological age. Such a result could stem from the fact that subjective age ratings are based on one’s experience while comparing it to a more general normative view of older adults—for example, how a person of such an age group should behave (Diehl et al., 2014). From a behavioral viewpoint, open individuals’ propensity to search for novel ideas and experiences might lead them to engage in a variety of healthy leisure, physical, and social behaviors more typical of younger adults’ habits than of their aged peers, or of the “stereotypical” older adult. As a result, open individuals might be more likely to feel younger, distancing themselves from their age group (Canada et al., 2013; Stephan et al., 2012). Openness also reflects a preference for novel and unconventional ideas and might therefore facilitate, from a psychological viewpoint, the adoption of a more flexible and counter-stereotypical view of one’s aging experiences that could contribute to a younger subjective age (Weiss et al., 2019). It is worth mentioning that Extraversion has also been often found to be associated with a youthful subjective age (Hubley and Hultsch, 1994, 1996; Stephan et al., 2012; Takatori et al., 2019); however, that was not the case here. The various ways of operationalizing felt age (difference between felt and chronological age instead of discrepancy score in Hubley and Hultsch, 1994, 1996; Stephan et al., 2012) and personality (the adjective check list in Takatori et al., 2019, instead of the BFI here) might account for such contrasting results.

Interestingly, personality made a larger additional contribution in explaining AARC-Gains and AARC-Losses scores. In line with previous evidence (e.g., Dunsmore and Neupert, 2022; Wahl et al., 2013), higher Emotional Stability was associated with lower awareness of age-related losses, whereas greater Extraversion was linked to a higher awareness of age-related gains. Emotionally stable individuals, better equipped to handle stress and adversities and less prone to intensified negative affective responses (e.g., anxiety, worry), are more likely to experience losses or daily life negative experiences occurring with aging in a less prominent, memorable, and threatening way, or they might encounter them in less sensitive, negatively biased, emotionally reactive ways than less emotionally stable individuals (e.g., Dunsmore and Neupert, 2022; Rupprecht et al., 2019; Wettstein et al., 2022). On the other hand, the propensity for sociability, activity, assertiveness, and energy characteristics of extraverted individuals might lead them to perceive their aging experiences in positive ways and gain a heightened awareness of gain-related changes that come with aging (e.g., Wettstein et al., 2022). The associations between AARC-Gains and the personality traits of Conscientiousness and Openness found in previous evidence (Dunsmore and Neupert, 2022; Wettstein et al., 2022) did not emerge here, but such contrasting findings could be related to different sets of covariates (age, gender and arthritis in Dunsmore and Neupert, 2022) and the larger age-range samples (40–98 years in Wettstein et al., 2022) enrolled in previous studies.

It should be acknowledged that some sociodemographic and self-rated health outcomes also contributed to explain personal VoA to a different extent, depending on the VoA facet considered. In particular, chronological age emerged as being associated with felt age, with older individuals feeling younger than their chronological age, according to previous evidence (Pinquart and Wahl, 2021). None of the sociodemographic and self-rated health outcomes contributed to explain AARC-Gains. It is worth mentioning that evidence of associations between sociodemographic characteristics and AARC-Gains are mixed, with studies finding either older age and/or being male or older age, having higher education, and being female are associated with lower AARC-Gains (see Sabatini et al., 2023). Also, associations between AARC-Gains and psychological and physical health outcomes are mixed and less consistent than those found for AARC-Losses and other personal VoA facets (see Sabatini et al., 2020a). Cultural differences, along with other factors (e.g., aging stereotypes, coping strategies, social environment) not examined here, might more likely influence individuals’ perceptions of age-related gains (see Sabatini et al., 2023). Finally, older age and poorer self-rated health made a substantial contribution in explaining greater awareness of age-related losses. These results align with evidence of a greater salience of perceived losses with increased aging (Baltes, 1987) and of the impact of AARC-Losses on psychological and physical health outcomes in adulthood and older age (Sabatini et al., 2020a).

Notwithstanding these interesting results, some limitations should be acknowledged. First, this was a cross-sectional study spanning the second half of life; therefore, future research examining the link between personality and personal VoA from a more comprehensive adult lifespan perspective is warranted. Moreover, a longitudinal design would allow for examination of the mutual interconnections and bidirectional nature of the associations between personality, as well as other relevant sociodemographic and health outcomes, and VoA. It might indeed be also plausible that VoA, by influencing goal selection, behavior and activities (Diehl and Wahl, 2010), could influence personality development and changes over the life course (e.g., Kornadt et al., 2019; Stephan et al., 2015b). It is worth mentioning that we focused here only on the two global AARC-Gains and AARC-Losses scores, rather than considering also their subdomains, in order to limit multiple testing and for sake of comparability with previous studies-which mostly used the two broad AARC scales-. Nonetheless, it would be of interest in future studies to gain a more nuanced picture of the association between personality dispositions and AARC by also examining AARC-Gains and AARC-Losses subdomains. Future studies should also consider other VoA concepts capturing more general, stereotypical views and mindsets related to the aging process and older adults as a group, as well as other relevant antecedents (e.g., occupational and socioeconomic status, living conditions, and social environment) not included here (e.g., Shrira et al., 2022; Weiss et al., 2019), to better elucidate their potential interplay with personality dispositions. It is also worth mentioning that, although the personality scale used here allows for an easy, acceptable and less time-consuming assessment of personality traits, its reliability is questionable, and may thus not capture personality dispositions as comprehensively as full-length Big Five measures, thereby leading to discrepant results with respect to previous evidence. Using more classical Big Five measures is therefore warranted to confirm and gain a better understanding of the potential personality–VoA associations. By doing so, not only the role of broad personality traits, but also of their narrower dimensions or facets of personality, known to make a unique contribution to the depiction of the personality traits they are designed to reflect (e.g., McCrae, 2015), could be explored further. Nonetheless, our results support the notion that certain personality traits—particularly Openness, Emotional Stability and Extraversion—could make a modest though significant contribution in explaining some personal VoA dimensions, particularly those related to subjective age and awareness of age-related changes in midlife and older age. Personality’s role in VoA seems to depend, however, on the VoA measure used, confirming the importance of considering the multidimensional nature of this construct.

To conclude, this study further highlights how personality dispositions could impact the processing of daily life aging experiences, thereby also shaping individuals’ personal VoA in terms of sensitivity toward, evaluation of, and affective and behavioral reactions to the age-related changes they experience (Hubley and Hultsch, 1994, 1996; Rupprecht et al., 2019). Therefore, our results could offer insight for future research in the context of the recent theoretical framework of VoA (Shrira et al., 2022) and promote a more comprehensive understanding of the behavioral (e.g., healthier leisure and social aging lifestyles) and psychological (e.g., beliefs, emotion regulation strategies, coping styles) pathways, driven also by personality traits, linking VoA to various health domains and longevity outcomes. Considering personality among those individual predispositions capable of delineating individual profiles prone to hold negative VoA, and therefore good targets for interventions aimed at eradicating stereotypical, ageist, maladaptive VoA, could be of interest to promote active/healthy aging.

## Data Availability

The raw data supporting the conclusions of this article will be made available by the authors, without undue reservation.

## Footnotes

^1^We checked for outliers in felt age according to the typical approach used in previous studies (e.g., Stephan et al., 2015a). None of the participants had felt age scores below 3 standard deviations, but we found three participants feeling older than their chronological age whose scores were slightly above 3 standard deviations (0.26) from the mean (one 0.29 and two 0.32). Evidence suggests that extremely older proportional-discrepancy scores are not reported randomly, but rather reflect authentic, intentional evaluation, that could be considered within the distribution of the data (see Palgi et al., 2018). We therefore decided to consider these three participants in the analyses. It is to note that results did not change excluding them, and there was no evidence of influential case bias, as the Cook’s *D* values for felt age were all less than one.

^2^Predictor variables were adjusted by means of grand-mean centering, to improve the interpretation of the intercept values.
