# Supplementary material for: Personal views of aging in midlife and older age: the role of personality
Source: Front Psychol. 2024 Oct 9;15:1437232. doi: 10.3389/fpsyg.2024.1437232 (PMC11497127; doi:10.3389/fpsyg.2024.1437232)
Supplement: Supplementary file 1 [file Table_1.docx]

Supplemental materials

**Personal views of aging in midlife and older age: the role of personality**

**Table S1.** *Matrix of correlations among the outcome measures of interest.*

|  | 1 | 2 | 3 | 4 | 5 | 6 | 7 | 8 | 9 | 10 | 11 |
| --- | --- | --- | --- | --- | --- | --- | --- | --- | --- | --- | --- |
| 1. Age | -- |  |  |  |  |  |  |  |  |  |  |
| 2. Gender | .05 | -- |  |  |  |  |  |  |  |  |  |
| 3. Education | -.39^**^ | -.01 | -- |  |  |  |  |  |  |  |  |
| 4. Self-rated health | -.11 | .22^**^ | .24^**^ | -- |  |  |  |  |  |  |  |
| 5. Agreeableness | .02 | .07 | -.01 | .07 | -- |  |  |  |  |  |  |
| 6. Conscientiousness | .02 | -.10 | -.01 | .07 | -.02 | -- |  |  |  |  |  |
| 7. Emotional Stability | -.07 | .16^*^ | .12 | .36^**^ | .08 | .00 | -- |  |  |  |  |
| 8. Extraversion | .06 | .03 | -.05 | -.03 | .01 | .05 | .04 | -- |  |  |  |
| 9. Openness | -.13^*^ | .01 | .21^**^ | .11 | .09 | .05 | .06 | .16^*^ | -- |  |  |
| 10. Felt age | -.15^*^ | -.09 | -.05 | -.15^*^ | -.10 | -.04 | -.17^*^ | .03 | -.17^*^ | -- |  |
| 11. AARC-Gains | .09 | -.05 | -.12 | .08 | .03 | .08 | .15^*^ | .22^**^ | -.00 | -.11 | -- |
| 12. AARC-Losses | .30^**^ | -.20^**^ | -.28^**^ | -.44^**^ | -.10 | -.11 | -.39^**^ | -.07 | -.19^**^ | .18^**^ | .16^*^ |

*Notes. Age: chronological age; Gender was a dichotomous variable (0=female, 1=male); AARC: Awareness of Age-Related Change; age: chronological age.*
